# A-770041 reverses paclitaxel and doxorubicin resistance in osteosarcoma cells

**DOI:** 10.1186/1471-2407-14-681

**Published:** 2014-09-19

**Authors:** Zhenfeng Duan, Jianming Zhang, Shunan Ye, Jacson Shen, Edwin Choy, Gregory Cote, David Harmon, Henry Mankin, Yingqi Hua, Yu Zhang, Nathanael S Gray, Francis J Hornicek

**Affiliations:** Center for Sarcoma and Connective Tissue Oncology, Massachusetts General Hospital, 100 Blossom St., Jackson 1115, Boston, 02114 MA USA; Cutaneous Biology Research Center, Massachusetts General Hospital, Boston, 02114 MA USA; Shanghai First People’s Hospital, Shanghai Jiaotong University, Shanghai, 200080 China; Department of Orthopedic Surgery, Liu Hua Qiao Hospital, Guangzhou, 510010 China; Dana Farber Cancer Institute, Harvard Medical School, Boston, MA 02115 USA

**Keywords:** Osteosarcoma, MDR, Src kinase, Doxorubicin

## Abstract

**Background:**

Reversing multidrug resistance (MDR) has been an important goal for clinical and investigational oncologists. In the last few decades, significant effort has been made to search for inhibitors to reverse MDR by targeting ATP-binding cassette (ABC) transporters (Pgp, MRP) directly, but these efforts have achieved little clinical success. Protein kinases play important roles in many aspects of tumor cell growth and survival. Combinations of kinase inhibitors and chemotherapeutics have been observed to overcome cancer drug resistance in certain circumstances.

**Methods:**

We screened a kinase specific inhibitor compound library in human osteosarcoma MDR cell lines to identify inhibitors that were capable of reversing chemoresistance to doxorubicin and paclitaxel.

**Results:**

We identified 18 small molecules that significantly increase chemotherapy drug-induced cell death in human osteosarcoma MDR cell lines U-2OS_MR_ and KHOSR_2._ We identified A-770041 as one of the most effective MDR reversing agents when combined with doxorubicin or paclitaxel. A-770041 is a potent Src family kinase (Lck and Src) inhibitor. Western blot analysis revealed A-770041 inhibits both Src and Lck activation and expression. Inhibition of Src expression in U-2OS_MR_ and KHOSR_2_ cell lines using lentiviral shRNA also resulted in increased doxorubicin and paclitaxel drug sensitivity. A-770041 increases the intracellular drug accumulation as demonstrated by calcein AM assay.

**Conclusions:**

These results indicate that small molecule inhibitor A-770041 may function to reverse ABCB1/Pgp-mediated chemotherapy drug resistance. Combination of Src family kinase inhibitor with regular chemotherapy drug could be clinically effective in MDR osteosarcoma.

**Electronic supplementary material:**

The online version of this article (doi:10.1186/1471-2407-14-681) contains supplementary material, which is available to authorized users.

## Background

Osteosarcoma, the most common primary malignant tumor of bone, is typically treated with surgery and adjuvant chemotherapy [1, 2]. Chemotherapy usually includes a combination of methotrexate, doxorubicin, and cisplatin. Unfortunately, the efficacy of these agents is hampered by the eventual development of multidrug resistance (MDR). This plateau in terms of overall survival has been present for a few decades without change. Almost one third of patients with localized osteosarcoma experience recurrent or progressive disease (usually caused by drug resistance) and the average survival period after a recurrence are about one year [1–3]. Therefore, overcoming MDR has been a high priority for both clinical and investigational oncologists [4–6]. Unfortunately, the mechanism of acquiring MDR in osteosarcoma is not well understood. A variety of mechanisms, including overexpression of the ABC drug efflux pumps P-glycoprotein (Pgp), and elevation of the apoptotic threshold that contributes to drug resistance [5, 7–12], have been proposed to play important roles for cancers acquiring MDR. Targeting multidrug resistance is one of the biggest challenges for successful treatment of osteosarcoma.

The human kinome contains approximately 600-protein kinases that mediate phosphorylation of proteins at an estimated 250,000 sites [13, 14]. Multiple lines of evidence indicate cross-talk between kinase mediated signaling pathways and multidrug resistant in different cancers. Several kinases such as IGF-1R, PI3K/AKT, PDGFR, mTOR and Src have been found to be highly expressed in different osteosarcomas, particularly in the late stage of the development of drug resistant tumor [15–17]. Inhibition of AKT enhances the cytotoxic effects of both paclitaxel and doxorubicin in tumor model systems [18]. Small interfering RNA (siRNA) downregulation of IGF-IR expression in MDR osteosarcoma cell lines also causes resensitization to doxorubicin [19, 20]. It has been shown that suppression of several kinases, such as PI3K/AKT/mTOR, Src, IGF-R, EGFR, JIK, Jak, or MEK/ERK significantly enhance cell death in the presence of low concentrations of chemotherapeutic drug, suggesting the potential utility of these kinases as drug targets [5, 13, 15, 19, 21]. Recently, some kinase inhibitors have also been found to reverse MDR by inhibiting the function of ABC transporters, including Pgp or MRP, or enhancing the efficacy of conventional chemotherapeutic drugs induced apoptosis [22, 23].

Based on these observations, it is anticipated that kinases represent a class of potential therapeutic targets in chemotherapy resistant osteosarcoma. We hypothesized that inhibiting of the expression and activation of certain kinases may enhance the efficacy of chemotherapy and/or reverse MDR. In order to identify the most suitable kinase candidates as therapeutic targets in drug resistant osteosarcoma patients, we screened a well-annotated kinase inhibitor focused library comprised of 3,000 commercially available known kinase inhibitors, as well as novel, ATP competitive kinase inhibitors targeting either active or inactive kinase conformations [24]. We systematically evaluated the potential reversal of MDR by the identified agent A-770041 and further studied the underlying mechanisms.

## Methods

### Kinase inhibitor library

We assembled a library of approximately 3000 diverse inhibitors and screened a subset of the library for kinome-wide selectivity profiling using binding (KinomeScan) [25], enzymatic (SelectScreen, Invitrogen Carlsbad, CA) and chemical proteomic approaches (Kinativ) [26]. This library is comprised of 500 commercially available known kinase inhibitors, as well as novel ATP competitive kinase inhibitors targeting either active or inactive kinase conformations. These compounds are characterized as being relatively potent and selective toward a relatively narrow array of kinase targets. This library was then screened against a number of different cancer cell lines or cells engineered to be associated with a defined oncogene (*BRFV600E*, *KRAS*, *c-Myc,* etc.) [27, 28].

### Human osteosarcoma cell lines

The multidrug resistant U-2OS_MR_ (established by selection against doxorubicin) and U-2OS_TR_ (established by selection against taxol/paclitaxel) was established in our laboratory as previously reported [20, 23]. The multidrug resistant KHOS_R2_ (established by selection with doxorubicin) cell line was kindly provided by Dr. Efstathios Gonos (Institute of Biological Research and Biotechnology, Athens, Greece) [29]. These cell lines were cultured in RPMI 1640 (Invitrogen, CA) supplemented with 10% FBS, 100 units/mL penicillin, and 100 μg/mL streptomycin (Invitrogen). Cells were incubated at 37°C in 5% CO_2_-95% air atmosphere and passaged when near-confluent monolayers were achieved using trypsin-EDTA solution. Drug resistant cell lines were periodically cultured in the respective drug to confirm their drug resistance characteristics. Cells were free from Mycoplasma contamination as tested using the MycoAlert Mycoplasma Detection Kit from Cambrex (East Rutherford, NJ).

### Drugs

Doxorubicin and paclitaxel were provided by the pharmacy at the Massachusetts General Hospital Cancer Center. The stock solutions of drugs were prepared according to the manufacturer’s specifications and stored at -20°C.

### Screening kinase inhibitor focused library

**Figure 1:**
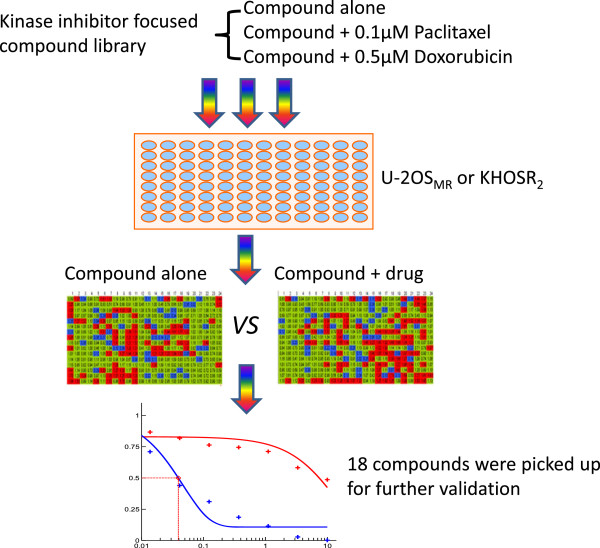
**Strategy for screening a kinase specific compound library in multidrug resistant osteosarcoma cell lines.** The kinase inhibitor screening was performed at the concentration of 0.66 μM and 1.8 μM with or without the presence of paclitaxel (0.1 μM) or doxorubicin (0.5 μM) in a four-day cellular proliferation assay. The combinatory drug effect was measured via CellTiter-Glo® Luminescent Cell Viability Assay Kit as described in Methods. Red: no effect on drug sensitivities; green: minor effect on drug sensitivities; blue: significant effect on drug sensitivities.

We screened the kinase specific compound library in human osteosarcoma MDR cell lines U-2OS_MR_ and KHOS_R2_ to determine the role of kinases in supporting chemoresistance to doxorubicin and paclitaxel in these MDR cells. The kinase inhibitor screening was performed at the concentration of 0.66 μM and 1.8 μM with or without the presence of paclitaxel (0.1 μM), doxorubicin (0.5 μM) in a four-day cellular proliferation assay (Figure 1). The combinatory drug effect was measured via CellTiter-Glo® Luminescent Cell Viability Assay Kit (Promega, Madison, WI) for automated high-throughput screening (HTS), cell proliferation and cytotoxicity assays.

### MTT cell proliferation assay

Drug cytotoxicity was assessed *in vitro* using the MTT assay as previously described [15, 23]. Briefly, 2 × 10^3^ cells per well were plated in 96-well plates in culture medium (RPMI 1640 supplemented with 10% FBS and penicillin/streptomycin) containing increasing concentrations of either A-770041, doxorubicin, paclitaxel alone or in combination with both. After 96 h of culture, 20 μL of MTT (5 mg/mL in PBS, purchased from Sigma) were added to each well and the plates were incubated for 4 h. The resulting formazan product was dissolved with acid (HCL)-isopropanol and the absorbance at a wavelength of 490 nm (A490) was read on a SPECTRAmax Microplate Spectrophotometer (Molecular Devices). Experiments were done in triplicate. Dose–response curves were fitted using GraphPad PRISM 4 software (GraphPad Software).

### Lentiviral Src kinase shRNA transduction

The effect of Src kinase knockdown on drug sensitivity in osteosarcoma MDR cell lines U-2OS_MR_ and KHOS_R2_ was examined using MISSION® LentiExpress™ human Src kinases shRNA (Sigma, St Louis, MO, TRC number: TRCN0000038149, Target sequence: 5′-GCTCGGCTCATTGAAGACAAT -3″). Transduction was carried out by following the manufacturer’s protocol as previously described [30]. In brief, on day 1, U-2OS_MR_ or KHOS_R2_ cells were diluted to 2 × 10^4^cells/ml in complete medium. Polybrene (hexadimethrine bromide) was added to a final concentration of 11.3 μg/ml and then 90 μl of cell suspension was added to each well of a 96-well plate following by add 10 μl of Src kinases shRNA lentivirus solution and incubated overnight. On day 2, the media was gently aspirated and 100 μl of complete media with different concentrations of paclitaxel or doxorubicin were replaced in each well. On day 5, the number of viable cells was determined by MTT assay as described above.

### Western blotting

The human phosphorylated Lck (pLck), pSrc, pAKT (threonine [Thr]308), pmTOR, CDK11, survivin, and BcL-X_L_ antibodies were purchased from Cell Signaling Technologies (Dedham, MA). The Pgp monoclonal antibody C219 was purchased from Covance Inc. (Formerly Signet, Dedham, MA). The mouse monoclonal antibody to human actin was purchased from Sigma-Aldrich (St. Louis, MO). Western blot analysis was performed as previously described with modifications [30]. Briefly, the cells were lysed in 1X radioimmunoprecipitation assay (RIPA) lysis buffer (Upstate Biotechnology, Lake Placid, NY), and protein concentration was determined by the DC Protein Assay (Bio-Rad, Hercules, CA) with a spectrophotometer (Beckman DU-640, Beckman Instruments, Inc.). Total protein (25 μg) was resolved on NuPage 4% to 12% Bis-Tris gels (Invitrogen). After electrophoresis, proteins were transferred to PROTRAN® nitrocellulose transfer membranes (Whatman GmbH, Germany). Membranes were blocked for 2 hours at 4°C with Odyssey Blocking Buffer (LI-COR Biosciences, Lincoln, NE), then incubated at 4°C overnight with primary antibodies diluted in Odyssey Blocking Buffer. After incubating with primary antibodies, the membranes were washed with TBS-T (containing 0.1% Tween 20) three times for 5 minutes. The membranes were then incubated with IRDye800CW-conjugated goat anti-rabbit IgG or IRDye680-conjugated goat anti-mouse IgG secondary antibodies (LI-COR Biosciences) diluted in Odyssey Blocking Buffer for 1 hour at room temperature with shaking. The blots were then washed three times with TBS-T and rinsed again with PBS. The levels of expressed proteins were visualized by scanning the membrane on an Odyssey Infrared Imaging System (LI-COR Biosciences) with both 680- and 800-nm channels.

### Apoptosis assay

Apoptosis was evaluated by a caspase-cleaved keratin 18 based quantification kit, the M30-Apoptosense ELISA assay, as per manufacturer’s instructions (Peviva AB, Bromma, Sweden). The ELISA apoptosis detects a 21-kDa fragment of cytokeratin 18 that is only revealed after caspase cleavage of the protein. Osteosarcoma MDR cells of U-2OS_MR_ and KHOS_R2_ were seeded at 8 × 10^3^ cells/per well in a 96-well plate for 24 hours before being treated with 0.1 μM doxorubicin plus different concentrations of A-770041 for 48 hours. The cells were then lysed by adding 10 μl 10% NP-40 per well, and the manufacturer’s instructions for the apoptosis assay were followed. Apoptosis was also evaluated by Western blot using whole-cell lysates immunoblotted with specific antibodies to PARP (Cell Signaling Technologies) and its cleavage products.

### Intracellular accumulation of calcein AM

Osteosarcoma MDR cells of KHOS_R2_ were plated onto 96-well plates at a density of 4 × 10^3^ cells/well in a volume of 100 μl RPMI1640 medium and grown for 24 h. Different concentrations (0, 0.1, 0.5 and 1 μM) of A-770041 were added for one hour. Calcein AM (Invitrogen) was diluted to 1 μM in culture medium, and then 50 μl of calcein AM was added to each microplate well containing 100 μl of the culture medium. After 30 minutes of exposure to calcein AM at 37°C, the medium was removed by aspiration, and cells were counterstained with Hoechst 33342 (1 μg/ml in culture medium) for 2 min. The intracellular accumulation of calcein AM was then visualized and quantified on a Nikon Eclipse Ti-U fluorescence microscope equipped with a SPOT RT digital camera.

### Statistical analysis

Statistical analyses were performed using the GraphPad PRISM5 software from GraphPad Software, Inc (San Diego, CA, USA). The student t test was used to analyze the differences between two groups. Results are expressed as mean ± SD and P < 0.05 was considered statistically significant.

## Results

### Identification of kinase inhibitors that reverse drug resistance in osteosarcoma MDR cell lines

After screening 3,000 compounds from a preselected, kinase-based small molecule library, we identified 18 small molecule compounds that can significantly increase chemotherapy drug-induced cell death in human osteosarcoma cell lines U-2OS_MR_ and KHOS_R2_ (Additional file 1: Table S1). Several previously reported kinase inhibitors such as dasatinib, AP25434, GSK461364, GP74514A and JNK-IN-X were also on the list of 18 small molecule compounds. We further verified efficacy of those 18 lead compounds by serially titrating drug combination studies with doxorubicin and paclitaxel. Eight of them showed improved IC_50_ in combination with doxorubicin or paclitaxel. These 8 small molecule compounds include inhibitors targeting multiple pan-tyrosine kinases such as Src family kinase (A-770041), kinases in cell cycle regulations such as PLK1 (GSK461364), CDK (GP74514A), and kinases in cell stress responses such as JNK (JNK-IN-X). Further studies validated A-770041, a compound previously reported as a highly potent pan-Src family kinase inhibitor [31–33], as the top one of the most effective MDR reversing agents when used in combination with doxorubicin or paclitaxel, as determined by drug sensitivity assays. Because of their target specificity and wide range coverage of the human kinome, the kinase inhibitor focused library compounds were anticipated to be useful to more easily identify the kinase mediators of cancer cell survival associated signaling that could be exploited for the purpose of drug development. Identified drug candidates could ideally provide further insight into the mechanism(s) involved in cancer MDR development, and at the same time potentially aid in developing and optimizing novel small molecule inhibitors able to overcome drug resistance and kill cancer cells more effectively.

### Structure of A-770041 and its activities reversing drug resistance

**Figure 2:**
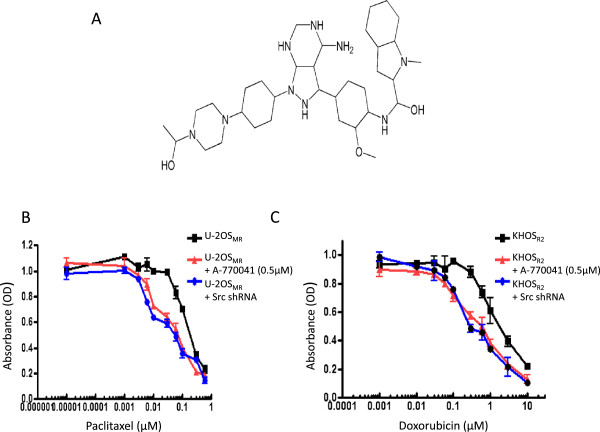
**A-770041 or Src shRNA reverses drug resistance. (A)** Structure of A-770041. **(B)** A-770041 or Src lentiviral shRNA reverse paclitaxel resistance inU-2OS_MR_ cell line. **(C)** A-770041 or Src lentiviral shRNA reverse doxorubicin resistance in KHOS_R2_ cell line. Results are expressed as mean ± standard deviations (SD).

A-770041 was reported as an inhibitor Lck, a Src-family kinase expressed in lymphocytes [31, 32]. We examined the effect of A-770041 on increasing the chemotherapy sensitivities of osteosarcoma MDR cell lines. After exposing the U-2OS_MR_ or KHOS_R2_ cell lines to paclitaxel, doxorubicin, or A-770041 alone, or the combination of paclitaxel or doxorubicin with A-770041 in complete cell culture medium for 96 hours, the relative numbers of viable cells were determined by MTT assay. Incubation of both U-2OS_MR_ and KHOS_R2_ cell lines with nonlethal concentrations of A-770041 (0.5 μM) was found to increase paclitaxel and doxorubicin drug sensitivities and reverse drug resistance (Figure 2).

### Effects on drug sensitivities from inhibiting Src expression by shRNA

A-770041 is known as a Src-family kinase inhibitor. To evaluate the contribution of Src mRNA expression levels to drug resistance in osteosarcoma MDR cell lines, the Src expression in U-2OS_MR_ or KHOS_R2_ cells was inhibited by using lentiviral Src kinase shRNA. The relative paclitaxel or doxorubicin sensitivities were determined by MTT in Src shRNA-transduced and in control MDR cell lines. Cytotoxicity was measured 96 hours after transduction with Src shRNA and treatment with paclitaxel or doxorubicin (see Methods). The results showed that Src down-regulation by shRNA partially recovered sensitivity to paclitaxel and doxorubicin (Figure 2B and Figure 2C).

### A-770041 is synergistic with paclitaxel and doxorubicin in KHOS_R2_ cell lines with ABCB1/Pgp overexpression

**Figure 3:**
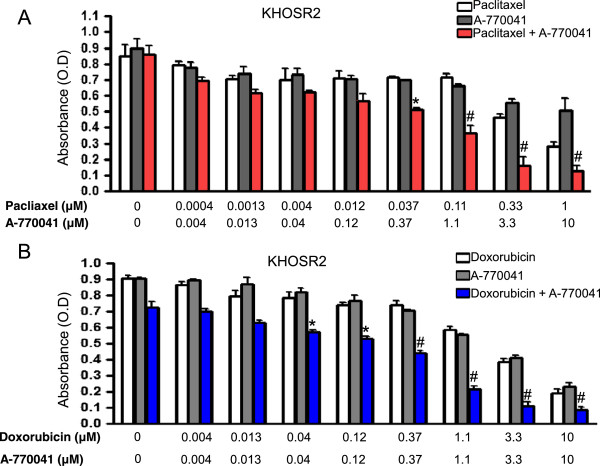
**Synergistic effect of A-770041 with paclitaxel or doxorubicin in drug resistant cell line.** The KHOS_R2_ cells were cultured in the presence of varying concentrations of either paclitaxel (0.0004–1 μM, **A)** or doxorubicin (0.004–10 μM, **B)** alone or in combination with A-770041 (0.004–10 μM) in regular RPMI1640 medium for 96 hours. The inhibition of cell growth was determined by MTT assay. The results are shown as the mean value of triplicate samples and are representative of 3 independent experiments. The student t test was used to analyze the differences between two groups. *P < 0.01 *vs*. paclitaxel or A-770041 treated cells alone, #*P* < 0.001 *vs*. paclitaxel or A-770041 treated cells alone.

The potential reversal of MDR by A-770041 was further evaluated in KHOS_R2_ cell lines with defined overexpression of the major MDR transporter ABCB1/Pgp. This MDR osteosarcoma cell line is remarkably resistant to the corresponding transporter substrate anticancer drugs including paclitaxel and doxorubicin. Subsequently, the synergistic effects of combinations of A-770041 with paclitaxel or doxorubicin in different ratios were evaluated in KHOS_R2_ cell lines. Synergistic cytotoxic effects were found in combinations of A-770041 with paclitaxel or doxorubicin in KHOS_R2_ MDR cell lines (Figure 3). The sensitivity to A-770041 was also tested in a panel of non MDR osteosarcoma cell lines including U-2OS, KHOS, SaoS, MG63 cell lines. A-770041 alone was found to exhibit similar anticancer activity in these drug sensitive cell lines as in drug resistant U-2OS_MR_ or KHOS_R2_ cell lines (data not shown).

### Effect of A-770041 on the expression and activation of Src, Lck

**Figure 4:**
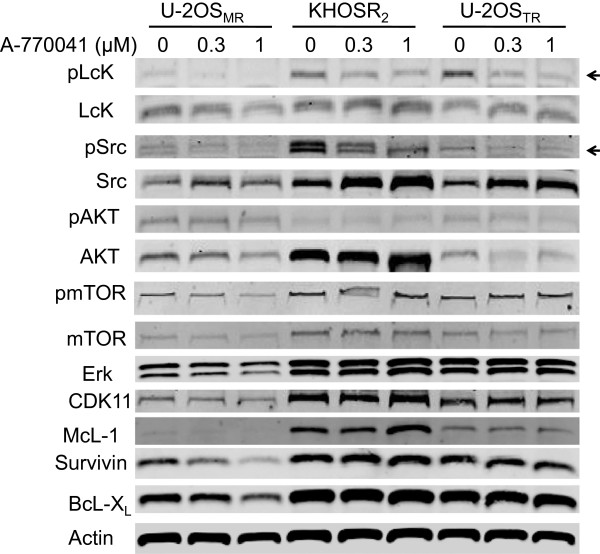
**A-770041 inhibits Src and Lck expression in drug-resistant osteosarcoma cells in a dose-dependent manner.** U-2OS_MR_, KHOS_R2_ or U-2OS_TR_ cells were treated with A-770041 (0, 0.3, or 1 μM ) in regular RPMI1640 medium for 48 hours. Total cellular proteins were subjected to Western blotting with specific antibodies as described in Methods. The levels of expressed proteins were visualized by scanning the membrane on an Odyssey Infrared Imaging System. Arrows indicate the confirmation of A-770041 inhibits both Src and Lck activation and expression as determined by densitometric analysis of Western blot results.

Src kinase expression and activation has been shown as the primary pathway involved in the malignant osteosarcoma phenotype [34, 35]. The effects of A-770041 on the expression of pSrc, pLck, pAKT and other kinases or apoptotic proteins in osteosarcoma MDR cell lines were analyzed by Western blot. The U-2OS_MR_, KHOS_R2_ or U-2OS_TR_ cell lines were incubated either with a range of concentrations (0, 0.3 or 1 μM) of A-770041 for 48 hours. Western blot analysis revealed that A-770041 inhibits both Src and Lck activation and expression in osteosarcoma MDR cells, but has less or no effect on other kinases such as pAKT, pmTOR or CDK11 (Figure 4). Treatment with A-770041 also decreased expression of the antiapoptotic proteins survivin and Bcl-X_L_ in the U-2OS_MR_ cell line (Figure 4).

### A-770041 induced apoptosis contributes to the reversal of Pgp-mediated paclitaxel and doxorubicin resistance

**Figure 5:**
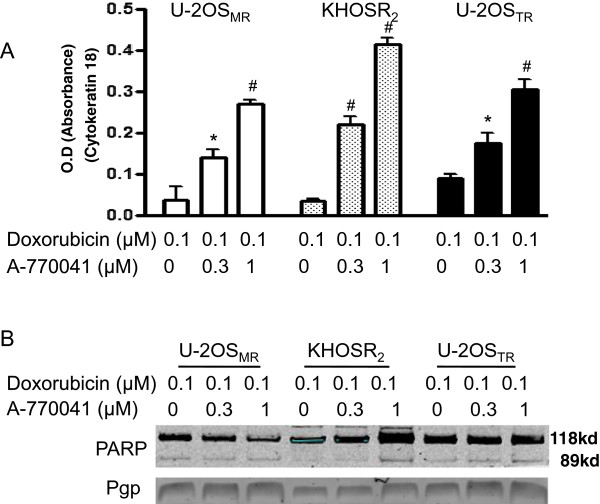
**A-770041 enhances apoptosis induced by doxorubicin in drug resistant osteosarcoma cells. (A)** U-2OS_MR_, KHOS_R2_ or U-2OS_TR_ cells were seeded at 8 × 10^3^ cells/per well in a 96-well plate for 24 hours before being treated with 0.1 μM doxorubicin plus different concentrations of A-770041 for 48 hours. The cells were lysed with 10% NP40, and the M30-Apoptosense ELISA assay was done as described in Methods. Results are expressed as mean ± SD. The student t test was used to analyze the differences between two groups. *P < 0.01 *vs*. doxorubicin treated cells alone, # P < 0.001 *vs*. doxorubicin treated cells alone. **(B)** Apoptosis was also evaluated by Western blot using whole-cell lysates immunoblotted with specific antibodies to PARP.

Since the combination of A-770041 with paclitaxel or doxorubicin is highly synergistic (Figure 3), we also investigated whether A-770041 possesses properties other than Pgp inhibition to reverse Pgp-mediated resistance. The U-2OS_MR_ or KHOS_R2_ cell lines were treated with a combination of low concentration of doxorubicin (0.1 μM) and A-770041 (0.3 μM, 1 μM) for 48 hours, after which the extent of apoptosis was measured. While doxorubicin only led to insignificant apoptosis at this low concentration, its combination with A-770041 was found to dramatically increase the apoptosis in the resistant U-2OS_MR_ or KHOS_R2_ cells as demonstrated by M30-Apoptosense ELISA assay (Figure 5A). The effect of A-770041 on the induction of apoptosis was further investigated by immunoblotting for PARP cleavage. PARP cleavage was detected after the treatment of U-2OS_MR_ or KHOS_R2_ cells with A-770041 in combination with doxorubicin (Figure 5B). In addition to PARP cleavage assay, quantification of apoptosis was also evaluated by checking Caspase-3/7 activations. Compared with cells treated with doxorubicin alone, the combination of A-770041 resulted in greater levels of apoptosis in these osteosarcoma MDR cell lines (data not shown). The reversal of Pgp-mediated drug resistance by A-770041 may also be associated with alteration of the transporter expression. Therefore, protein expression of Pgp was examined in U-2OS_MR_, KHOS_R2_ and U-2OS_TR_ cells. After incubating the cells with A-770041 at concentrations up to 1 μM for 48 hours, A-770041 did not alter the expression of Pgp protein levels (Figure 5B).

### A-770041 modulates Pgp-mediated uptake and efflux of calcein AM

**Figure 6:**
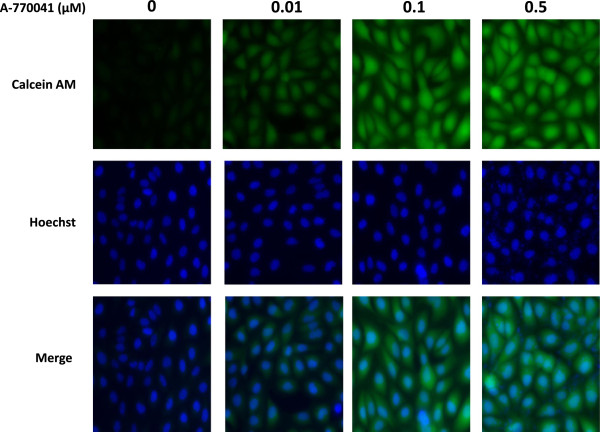
**Fluorescent images of dose-dependent increase in intracellular accumulation of calcein AM in KHOS**_**R2**_ **following 30 min treatment with A-770041.** The calcein AM assay was optimized and performed using the Vybrant Multidrug Resistance kit in KHOS_R2_ cells. Cells were plated onto 96-well plates at a density of 4 × 10^3^ cells/well in a volume of 100 μl RPMI1640 medium and grown for 24 h. Different concentrations (0, 0.01, 0.1 and 0.5 μM) of A-770041 were added for one hour. 50 μl of calcein AM were added to each well. After 30 minutes exposure to calcein AM at 37°C, cells were counterstained with Hoechst 33342 for 2 min. The cell fluorescence images were acquired by a fluorescence microscope.

Reversing Pgp-mediated MDR will result in an intracellular accumulation of chemotherapeutics, which can be achieved by inhibiting Pgp function. Therefore, we examined the effect of A-770041 on the uptake and efflux of Calcein AM, a substrate of Pgp in KHOS_R2_. Pgp inhibition can be directly correlated with the amount of intracellular Calcein AM fluorescence. A-770041 was shown to increase intracellular accumulation of Calcein in osteosarcoma MDR cell line KHOS_R2_ in a dose-dependent manner as determined by image analysis (Figure 6). A-770041 had a prominent effect on the accumulation of Calcein in KHOS_R2_ cells at a concentration as low as 0.01 μM. In the control parental drug-sensitive cell lines U-2OS and KHOS, which do not overexpress Pgp, A-770041 had no evident effect on accumulation of Calcein (data not shown).

## Discussion

Protein kinases play important roles in the many aspects of cancer including in maintaining or supporting the drug resistance phenotype [13, 22, 23]. Overexpression and activation of specific kinases have been associated with progressive drug resistance. Several recent reports have demonstrated that some kinase inhibitors or other agents are also potent inhibitors of Pgp or MRP transporters and thereby increase drug sensitivities [22, 23, 36–39]. Synergistic cytotoxic effects of inorganic phosphate and chemotherapeutic drugs on human osteosarcoma cells have been reported recently [38, 39]. Small molecular compounds capable of inhibiting specific kinases and Pgp represent a desirable approach for overcoming Pgp-mediated drug resistance in human cancer. In this study, we report that A-770041, a known Src family kinase inhibitor originally developed as an immunosuppressant to prevent acute graft rejection [31–33], is a novel MDR reversing agent whose mechanism of action includes both the inhibition of Src and Lck activation and expression, and the inhibition of Pgp function by increases the intracellular drug accumulation.

Cell-based screening of a preselected kinase-based small molecule compound library identified A-770041 as one of the most effective drug resistance reversing agents in two osteosarcoma MDR cell line models, U-2OS_MR_ and KHOS_R2,_ having defined overexpression of the major MDR transporters, ABCB1/Pgp. These osteosarcoma resistant cell lines have been extensively characterized with stable MDR phenotype in previous studies [6, 7, 20, 29]. MTT assay showed A-770041 significantly overcomes drug resistance in both U-2OS_MR_ and KHOS_R2_ MDR cell lines. Knockdown of Src with lentiviral shRNA also increased drug sensitivity to transporter substrate anticancer drugs paclitaxel and doxorubicin, suggesting that blocking the Src pathway might overcome MDR in osteosarcoma. We then evaluated A-770041’s potential synergistic cytotoxic effect when used in combination with paclitaxel and doxorubicin. The data showed that A-770041 is highly synergistic with both paclitaxel and doxorubicin in these MDR cell lines. As demonstrated by Western analysis, part of these effects was potentially through decreased expression of Src and Lck.

Src is a nonreceptor tyrosine kinase encoded by the Src proto-oncogene. It is one of the eight members (Src, Fyn, Yes, Lck, Lyn, Hck, Fgr and Blk) of the Src family of kinases. Both overexpression and overactivation of Src have been shown to aid in the development of osteosarcoma by promoting key oncogenic mechanisms such as cell proliferation, adhesion, invasion, and resistance to apoptosis induced by chemotherapy drugs [34, 40, 41]. Src kinase activity is regulated via a variety of receptor and non-receptor tyrosine kinases such as EGFR, PDGF, and Jak. Activation of Src kinase leads in turn to activation of a variety of downstream signaling pathways such as the Ras/ MAPK pathway, and activation of the Stat3, resulting in cell cycle progression from G2 to M phase and VEGF production, which aids in angiogenesis and tumor growth and invasion. Src is highly expressed and activated in sarcomas of diverse subtypes, including high-grade osteosarcoma, leiomyosarcoma, synovial sarcoma and liposarcoma [34, 40]. The Src inhibitor dasatinib (BMS-354825) induces apoptosis in osteosarcoma cells [35]. A more recent study showed treatment of synovial sarcoma cells with dasatinib also led to apoptosis, decreased proliferation, and was associated with reduced phosphorylation of IGF-1R, AKT and Stat3. Furthermore, combination treatment with dasatinib and chemotherapeutic agents results in additive effects [40]. Inhibition of Src protein expression by small interfering RNA also induced apoptosis [35]. Inhibition of Src and its downstream signaling pathways has been shown to inhibit growth, migration, and invasion of a variety of human cancer cells including osteosarcoma [35, 42]. It has been shown that inhibition of Src, either pharmacologically by Src inhibitor or through expression of a Src dominant-negative fusion construct, increased the cytotoxicity of paclitaxel or cisplatin, in both mouse and human cancer cells [21, 40, 42]. Importantly, Src inhibition has also been shown to reverse drug resistance in ovarian cancer MDR cells [42]. Cytotoxicity in response to Src inhibition was associated with enhanced apoptosis in these studies. Consistent with these findings, A-770041 also increased the apoptosis induced by doxorubicin in osteosarcoma MDR cell lines. The synergistic activity of A-770041 with doxorubicin suggests that A-770041 could induce apoptosis in doxorubicin-resistant cells through mechanisms independent of inhibition of Pgp.

The interaction of kinase inhibitor with ABC transporter Pgp has been documented in several studies. For example, gefitinib (Iressa), a selective EGFR tyrosine kinase inhibitor, is used for the treatment of lung cancer. It has been found that clinically achievable levels of gefitinib moderately reversed the Pgp-mediated resistance to paclitaxel and docetaxel in Pgp overexpressing cells [43]. Gefitinib increased the intracellular accumulation of the Pgp substrate rhodamine-123 in resistant cells, and activated ATPase in a preparation of pure Pgp-expressing membrane [43, 44]. These findings suggest that gefitinib may directly interact with Pgp and inhibit its function. Our current study showed A-770041 significantly increases intracellular accumulation of calcein in osteosarcoma MDR cell line KHOS_R2_ in a dose-dependent manner. These results indicated that A-770041 can also inhibit Pgp efflux function via interaction with Pgp. Drugs interacting with MDR transporter proteins like Pgp may be useful not only for the reversal of cancer drug resistance, but also for increasing the absorption or the brain entry of various pharmacological agents [22].

In summary, we found that A-770041 could increase the sensitivity of drug-resistant osteosarcoma cells to various cytotoxic chemotherapeutic agents (doxorubicin, paclitaxel). These studies provided a proof of principle that A-770041 is active against osteosarcoma cell lines with known resistance to conventional anticancer agents, as well as primary tumor cells from osteosarcoma patients. Further *in vivo* research is warranted to understand the implication of A-770041 in overcoming drug resistance.

## Conclusions

Kinases play an essential role in cancer cell growth, survival and drug resistance, and these enzymes are actively investigated by the pharmaceutical industry to create molecular therapies.

In this study, a cell-based screening assay using the MDR osteosarcoma cell line was used to screen a kinase specific inhibitor compound library and identify small-molecule compounds capable of reversing MDR. We identified A-770041, a potent Src family kinase (Lck and Src) inhibitor, as one of the most effective MDR reversing agents when combined with doxorubicin or paclitaxel. Because osteosarcoma is characterized by frequent relapse that is associated with the development of MDR, these findings provide support for further development of A-770041 or its derivates as candidate inhibitors of MDR in osteosarcoma treatment.

## Electronic supplementary material

Additional file 1: **Identification of 18 kinase inhibitors that reverse drug resistance in osteosarcoma MDR cell lines.** (XLSX 12 KB)

## Abbreviations

MDR: Multidrug resistance
Lck: Lymphocyte-specific protein tyrosine kinase
Src: Src kinase.
